# Development and evaluation of a patient-reported outcome measure specific for Gaucher disease with or without neurological symptoms in Japan

**DOI:** 10.1186/s13023-023-02996-9

**Published:** 2024-01-05

**Authors:** Aya Narita, Yuta Koto, Shinichi Noto, Masafumi Okada, Midori Ono, Terumi Baba, Rieko Sagara, Norio Sakai

**Affiliations:** 1 Division of Child Neurology, Faculty of Medicine, Institute of Neurological Science, Tottori University, Yonago, Japan; 2 School of Nursing, Faculty of Health Science, Osaka Aoyama University, Osaka, Japan; 3 Department of Rehabilitation, Niigata University of Health and Welfare, Niigata, Japan; 4 Real-World Evidence Solutions & HEOR, IQVIA Solutions Japan K.K., Tokyo, Japan; 5 Japan Medical Office, Takeda Pharmaceutical Company Limited, Tokyo, Japan; 6 Child Healthcare and Genetic Science Laboratory, Division of Health Sciences, Osaka University Graduate School of Medicine, 1‑7 Yamadaoka, Suita, Osaka, 565‑0871 Japan

**Keywords:** Gaucher disease, Japan, Patient reported outcome measures, Quality of life, Burden of disease, Neuronopathic Gaucher disease, Reliability, Validity

## Abstract

**Background:**

Patients with Gaucher disease (GD), a rare lysosomal storage disorder, have reduced health-related quality of life (HRQOL). A patient-reported outcome measure (PROM) for HRQOL developed for type 1 GD (GD1) is not appropriate for patients with neuronopathic GD (nGD) types 2 (GD2) and 3 (GD3). In this study, we developed a new PROM for use in all GD types. We previously reported the qualitative analysis of interviews with Japanese patients with nGD, which was used to create nGD-specific PROM items. Here we evaluated the full PROM combining the type 1 questionnaire with the new nGD-specific items.

**Methods:**

Patients with confirmed GD were recruited (Association of Gaucher Disease Patients in Japan or leading doctors) for pre-testing (May 2021) or the main survey (October–December 2021). The PROM had three parts: Parts 1 and 2 were translated into Japanese from the pre-existing GD1 PROM, whereas Part 3 was newly developed. Patients (or their caregivers, where necessary) completed the PROM questionnaire on paper and returned it by mail. Mean scores were determined overall and by GD type. Inter-item correlations, content consistency (Cronbach’s alpha), and test–retest reliability (Cohen’s kappa; main survey only, taken 2 weeks apart) were calculated.

**Results:**

Sixteen patients (three with GD1; six with GD2; seven with GD3) and 33 patients (nine with GD1; 13 with GD2; 11 with GD3) participated in the pre-test and main survey, respectively. All GD2 patients and one-third (6/18) of GD3 patients required caregivers to complete the questionnaire. Mean scores indicated that the burden was highest in GD2 and lowest in GD1. In the main survey, internal consistency was high (Cronbach’s alpha = 0.898 overall, 0.916 for Part 3), and test–retest reliability was high for Part 3 (kappa > 0.60 for 13/16 items) but low for Part 1 (kappa < 0.60 for 12/15 items).

**Conclusions:**

We have developed a flexible and reliable PROM that can be tailored for use in all types of GD and propose using Parts 1 and 2 for GD1, Parts 2 and 3 for GD2, and Parts 1, 2, and 3 for GD3.

**Supplementary Information:**

The online version contains supplementary material available at 10.1186/s13023-023-02996-9.

## Background

Gaucher disease (GD) is a rare, autosomal recessively inherited, lysosomal storage disorder with a worldwide prevalence of 0.7 to 1.75 per 100,000 [1]. In Japan, the prevalence is estimated to be 1 in 330,000 [2], and a total of 211 patients are estimated to have GD [3]. Typical symptoms of GD include fatigue, anemia, thrombocytopenia, and hepatosplenomegaly [4]. GD is categorized into three clinical phenotypes based on the presence or absence of neurological symptoms: type 1 GD (GD1; Mendelian Inheritance in Man [MIM] code #230800) is non-neuronopathic GD, whereas types 2 (GD2; MIM code #230900) and 3 (GD3; MIM code #231000) have neurological symptoms, including abnormal eye movements, seizure, and developmental delay, and are collectively known as neuronopathic GD (nGD) [4]. GD1 accounts for approximately 94% of cases in non-Japanese populations [5]. In contrast, the proportion of patients with nGD in Japan is higher compared with other countries, and GD2 and GD3 account for approximately 60% of GD cases [3, 6, 7]. GD2, the most severe and rapidly progressive form, has a stereotypical presentation and death in infancy [8]. However, the phenotypic heterogeneity of GD2 has been increasingly recognized in recent decades, and multiple therapeutic approaches have prolonged survival in these patients [8, 9]. GD3 is a chronically progressive form of nGD with symptoms occurring before 20 years of age; the clinical course is variable and more heterogeneous than GD2, with affected individuals requiring assistance to fulfill activities of daily life [4]. In Japan, patients with nGD receive the standard treatment, enzyme replacement therapy (ERT), similar to patients with GD1. In addition, patients with nGD receive supportive care such as tracheostomy, home ventilator use, and antiepileptic drugs, depending on their symptoms. These treatments allow patients with nGD to live at home for longer than was previously possible.

Rare genetic diseases such as GD are chronic, and patients experience not only physical symptoms, but also psychological and social consequences that adversely affect long-term quality of life. Patient-reported outcome measures (PROMs) have been developed as a method to obtain and quantify patients’ views of their functional ability, symptoms, and health-related quality of life (HRQOL) [10]. As well as being a tool for use in clinical trials, PROMs can be used in clinical practice to improve communications between patients and clinicians about their disease progression and treatment outcomes [11]. Previous studies have assessed HRQOL in patients with GD [12, 13]; however, these studies did not use GD-specific measures of HRQOL and included only patients with GD1. General HRQOL measures do not provide sufficient information, and may not be sensitive enough to detect changes resulting from treatment, because many of the characteristics of GD, such as bone and neurological symptoms, are wide-ranging and have substantial effects on long-term quality of life. Although a GD-specific PROM questionnaire was recently developed for GD1 [14, 15], there is no PROM questionnaire that contains items on neurological symptoms or is specific for nGD. Therefore, a disease-specific measure of HRQOL for patients with GD, including nGD, is needed to assess the effectiveness of treatment and support.

The aim of this study was to develop a new PROM questionnaire that can be used to evaluate the burden of GD in all subtypes. In the first stage of the development of the PROM questionnaire, qualitative analysis of transcripts from interviews with Japanese patients with nGD and/or their caregivers was conducted [16]. The analysis included creating a coding list using text-mining software, followed by hierarchical cluster analysis and co-occurrence network analysis to identify keywords, major themes, and specific topics related to disease burden [16]. The results of these analyses were used to create a new set of PROM items specific for neurological symptoms that could be used in conjunction with a Japanese language–validated version of the existing GD PROM [14]. Here we report the subsequent development and evaluation of the combined PROM questionnaire, consisting of pre-testing followed by validation of the PROM in the main survey of Japanese patients.

## Results

### Pre-test

#### Demographic and baseline clinical characteristics

A total of 16 patients with GD (three with GD1, six with GD2, and seven with GD3) participated in the pre-test (Table 1). The mean age at diagnosis was 4.3, 1.0, and 6.6 years, and the mean current age was 46.3, 9.0, and 28.4 years in patients with GD1, GD2, and GD3, respectively. All three patients with GD1 and 4/7 (57.1%) patients with GD3 completed the PROM themselves, whereas all six patients with GD2 required a proxy to complete the PROM.

**Table 1:** Patient demographics and clinical characteristics

Characteristic	Pre-test				Main analysis			
	GD1 (N = 3)	GD2 (N = 6)	GD3 (N = 7)	Overall (N = 16)	GD1 (N = 9)	GD2 (N = 13)	GD3 (N = 11)	Overall (N = 33)
Sex, n (%)								
Male	1 (33.3)	2 (33.3)	3 (42.9)	6 (37.5)	2 (22.2)	8 (61.5)	6 (54.5)	16 (48.5)
Female	2 (66.7)	4 (66.7)	3 (42.9)	9 (56.3)	7 (77.8)	5 (38.5)	5 (45.5)	17 (51.5)
Not collected	0 (0)	0 (0)	1 (14.3)	1 (6.3)	0 (0)	0 (0)	0 (0)	0 (0)
Age at diagnosis, years								
Mean (SD)	4.3 (1.5)	1.0 (0.5)	6.6 (6.6)	4.1 (5.0)	23.4 (16.9)	0.6 (0.7)	8.1 (7.5)	9.3 (13.3)
Median (min, max)	4.0 (3.0, 6.0)	1.0 (0.2, 1.5)	3.0 (1.0, 18.0)	1.8 (0.2, 18.0)	24.0 (2.0, 60.0)	1.0 (0, 2.0)	5.0 (1.0, 24.0)	3.0 (0, 60.0)
Current age, years								
Mean (SD)	46.3 (3.5)	9.0 (7.9)	28.4 (18.1)	24.5 (18.8)	58.6 (18.5)	10.0 (8.2)	37.1 (16.9)	32.3 (24.6)
Median (min, max)	46.0 (43.0, 50.0)	7.5 (2.0, 23.0)	32.0 (4.0, 57.0)	23.5 (2.0, 57.0)	63.0 (25.0, 89.0)	9.0 (1.0, 31.0)	38.0 (10.0, 58.0)	31.0 (1.0, 89.0)
Duration of GD, years								
Mean (SD)	42.0 (2.0)	8.1 (7.9)	21.9 (13.2)	20.5 (15.7)	56.6 (27.0)	9.6 (8.1)	34.6 (17.9)	25.5 (23.2)
Median (min, max)	42.0 (40.0, 44.0)	7.0 (0.7, 22.0)	23.0 (2.3, 39.0)	22.5 (0.7, 44.0)	56.2 (25.0, 88.8)	8.8 (0.7, 30.1)	35.2 (9.8, 57.3)	16.7 (0.7, 88.8)
Not collected, n (%)	0 (0)	0 (0)	0 (0)	0 (0)	5 (55.6)	0 (0)	2 (18.2)	7 (21.2)
Respondent, n (%)								
Self	3 (100)	0 (0)	4 (57.1)	7 (43.8)	9 (100)	0 (0)	8 (72.7)	17 (51.5)
Proxy	0 (0)	6 (100)	3 (42.9)	9 (56.3)	0 (0)	13 (100)	3 (27.3)	16 (48.5)
Father	–	–	–	–	0 (0)	2 (15.4)	0 (0)	2 (6.1)
Mother	–	–	–	–	0 (0)	11 (84.6)	3 (27.3)	14 (42.4)

GD1/2/3: type 1/2/3 Gaucher disease; max: maximum; min: minimum; SD: standard deviation

#### PROM scores

The PROM questionnaire consisted of three parts (Additional file 1: Table S1; Additional file 2: Table S2): Parts 1 and 2 were translated into Japanese from the previously published PROM for GD1 [14], whereas Part 3 was newly developed. Part 1 assessed patient experiences over the previous month, whereas Parts 2 and 3 assessed experiences over the previous week.

For Part 1, mean (standard deviation [SD]) total scores were 27.5 (10.6) and 38.8 (27.2) for GD2 and GD3, respectively, out of a maximum of 150 (highest possible burden) (Table 2). Mean scores were not calculated for GD1 because data were not fully collected for all three patients. Data were also not fully collected for 4/6 (66.7%) and 3/7 (42.9%) patients with GD2 and GD3, respectively. In reviewing the answers to Item 14 (“Over the past month, all of my health concerns have been about Gaucher’s disease”), it appeared that the Japanese translation was misinterpreted as how large a burden GD was instead of the burden of other health concerns relative to GD. For this reason, the scores for this item were reversed for analysis. For the 15 individual items, the mean (SD) score in the overall pre-test population was lowest (0.625 [1.77]) for Part 1 Item 3 (P1-3) “Over the past month, my Gaucher disease has restricted my ability to have intimate relationships with my spouse/partner” and highest (6.00 [3.38]) for P1-14 “Over the past month, all of my medical concerns have been Gaucher-related” (Additional file 3: Table S3).

**Table 2:** PROM scores: summary of Parts 1, 2, and 3

Part	Pre-test				Main analysis			
	GD1 (N = 3)	GD2 (N = 6)	GD3 (N = 7)	Overall (N = 16)	GD1 (N = 9)	GD2 (N = 13)	GD3 (N = 11)	Overall (N = 33)
Part 1								
Mean (SD)	NA	27.5 (10.6)	38.8 (27.2)	35.0 (22.4)	42.1 (31.5)	13.8 (12.4)	65.0 (21.3)	45.0 (30.3)
Median (min, max)	NA	27.5 (20.0, 35.0)	30.0 (17.5, 77.5)	28.8 (17.5, 77.5)	37.5 (10.0, 97.5)	13.8 (5.00, 22.5)	65.0 (40.0, 90.0)	42.5 (5.00, 97.5)
Not collected, n (%)	3 (100)	4 (66.7)	3 (42.9)	10 (62.5)	3 (33.3)	11 (84.6)	7 (63.6)	21 (63.6)
Part 2								
Mean (SD)	17.0 (8.19)	45.8 (24.1)	16.2 (15.7)	25.5 (21.4)	17.0 (8.47)	38.7 (21.5)	26.5 (20.6)	29.1 (20.3)
Median (min, max)	15.0 (10.0, 26.0)	51.0 (12.0, 69.0)	8.00 (5.00, 45.0)	15.0 (5.00, 69.0)	17.0 (7.00, 28.0)	48.0 (7.00, 76.0)	28.0 (0, 67.0)	27.0 (0, 76.0)
Not collected, n (%)	0 (0)	2 (33.3)	1 (14.3)	3 (18.8)	1 (11.1)	0 (0)	0 (0)	1 (3.0)
Part 3								
Mean (SD)	NA	79.0 (41.4)	25.7 (12.7)	52.3 (40.0)	24.2 (16.5)	79.7 (42.5)	52.1 (30.2)	57.9 (39.6)
Median (min, max)	NA	95.0 (32.0, 110)	33.0 (11.0, 33.0)	33.0 (11.0, 110)	21.0 (5.00, 52.0)	94.5 (0, 126)	51.5 (13.0, 109)	52.5 (0, 126)
Not collected, n (%)	3 (100)	3 (50.0)	4 (57.1)	10 (62.5)	3 (33.3)	1 (7.7)	1 (9.1)	5 (15.2)

GD1/2/3: type 1/2/3 Gaucher disease; max: maximum; min: minimum; NA: not applicable; PROM: patient-reported outcome measure; SD: standard deviation

For Part 2, mean (SD) total scores were 17.0 (8.19), 45.8 (24.1), and 16.2 (15.7) for GD1, GD2, and GD3, respectively, out of a maximum of 90 (Table 2). For the nine individual items, the mean (SD) score in the overall pre-test population was lowest (1.53 [3.23]) for P2-2 “Over the past week, how visibly big or swollen has your abdomen looked because of your Gaucher disease?” and highest (5.69 [4.67]) for P2-1 “Over the past week, how dependent on others have you been because of your Gaucher disease?” (Additional file 3: Table S3).

For Part 3, mean (SD) total scores were 79.0 (41.4) and 25.7 (12.7) for GD2 and GD3, respectively, out of a maximum of 150 (Table 2). Mean scores were not calculated for GD1 because all three patients had data that were not fully collected. Data were also not fully collected for 3/6 (50.0%) and 4/7 (57.1%) patients with GD2 and GD3, respectively. For the 15 individual items, the mean (SD) score in the overall pre-test population was lowest (1.90 [2.69]) for the item “Over the past week, have you had any pain in the body?” (P3-6 in the pre-test, P3-7 in the main survey) and highest (5.90 [3.11]) for the item “Have you ever felt physically tired after visiting the hospital or treatment?” (P3-12 in the pre-test, P3-13 in the main survey) (Additional file 3: Table S3).

#### Cronbach’s alpha

Cronbach’s alpha, an indicator of content consistency, was calculated as 0.926 for the overall PROM, 0.841 for Part 1, 0.902 for Part 2, and 0.934 for Part 3.

#### Inter-item correlations

Inter-item correlations were mapped in a correlation matrix (Additional file 4: Figure S1). In general, most items in Part 2 were positively correlated with items in Part 3. P1-7 (“Because of my Gaucher disease, I am concerned I will be at risk of cancers”) was negatively correlated with other items in Part 1 and with several items in Parts 2 and 3, but positively correlated with P3-1 (“Over the past week, have you had any difficulty hearing?”). Most items in all three parts were negatively correlated with patient age and duration of disease.

#### Interviews

Interviews with the seven patients and nine caregiver proxies who completed the pre-test provided insights into the usefulness of the PROM for different GD types. For example, the items in Part 3 that focus on neurological symptoms are not applicable to GD1. In addition, patients with GD2 or GD3 who have cognitive or speaking difficulties were not able to convey their emotions to a proxy who completed the PROM; therefore, items related to emotions may not be useful for these patients.

The interviews also identified potential areas for improvement of the PROM. As a result of patient feedback and discussion among the authors, “swallowing food” and “speaking” were considered to be separate symptoms; therefore, the item “Over the past week, have you had any difficulty swallowing food or speaking?” was divided into two items in the main survey. Patients with all types of GD and/or their proxies found the 0–10 scale used in Parts 2 and 3 difficult to use; however, because the original English version of Part 2 used a 0–10 scale [14], we did not change the scale in either Part 2 or Part 3.

### Main survey

#### Demographic and baseline clinical characteristics

A total of 33 patients with GD (nine with GD1, 13 with GD2, and 11 with GD3) participated in the main survey (Table 1). About half (51.5%) of the patients were female. The mean age at diagnosis was 23.4, 0.6, and 8.1 years, and the mean current age was 58.6, 10.0, and 37.1 years in patients with GD1, GD2, and GD3, respectively. All patients with GD1 and 8/11 (72.7%) patients with GD3 completed the questionnaire themselves, whereas all patients with GD2 required a parent to complete the questionnaire as a proxy.

#### PROM scores

For Part 1, mean (SD) total scores were 42.1 (31.5), 13.8 (12.4), and 65.0 (21.3) for GD1, GD2, and GD3, respectively, out of a maximum of 150 (Table 2). Data were not fully collected for 3/9 (33.3%), 11/13 (84.6%), and 7/11 (63.6%) patients with GD1, GD2, and GD3, respectively, suggesting that the items in Part 1 were particularly difficult for patients with nGD to answer. In addition, many of the Part 1 items are not applicable to children with nGD (e.g. questions about how GD affects jobs, intimate relationships, financial burden, etc.). For the individual items, the mean (SD) score in the overall main survey population was lowest (1.17 [2.81]) for P1-3 “Over the past month, my Gaucher disease has restricted my ability to have intimate relationships with my spouse/partner” and highest (5.15 [2.65]) for P1-14 “Over the past month, all of my medical concerns have been Gaucher-related” (Additional file 3: Table S3), consistent with the pre-test findings.

For Part 2, mean (SD) total scores were 17.0 (8.47), 38.7 (21.5), and 26.5 (20.6) for GD1, GD2, and GD3, respectively, out of a maximum of 90 (Table 2). For the individual items, the mean (SD) score in the overall main survey population was lowest (1.03 [2.01]) in P2-2 “Over the past week, how visibly big or swollen has your abdomen looked because of your Gaucher disease?” and highest (5.12 [4.25]) in P2-1 “Over the past week, how dependent on others have you been because of your Gaucher disease?” (Additional file 3: Table S3), consistent with the pre-test findings.

For Part 3, mean (SD) total scores were 24.2 (16.5), 79.7 (42.5), and 52.1 (30.2) for GD1, GD2, and GD3, respectively, out of a maximum of 160 (Table 2). Data were not fully collected for 3/9 (33.3%), 1/13 (7.7%), and 1/11 (9.1%) patients with GD1, GD2, and GD3, respectively. Notably, the percentage of patients with nGD who could complete Part 3 was much higher in the main survey (90.9–92.3%) than in the pre-test (42.9–50.0%), indicating that the modifications to Part 3 made it easier for patients to complete. For the individual items, the mean (SD) score in the overall main survey population was lowest (1.94 [2.86]) in P3-11 “During the past week, have you had any concerns about continuing treatment for Gaucher's disease?” and highest (5.48 [3.44]) in P3-13 “Have you ever felt physically tired when you went to the hospital or after treatment?” (Additional file 3: Table S3). Scores for P3-13 were consistently high across GD subtypes, indicating that fatigue related to hospital visits and treatment was a burden for all patients with GD.

#### Cronbach’s alpha

Cronbach’s alpha was calculated as 0.898 for the overall PROM, 0.896 for Part 1, 0.882 for Part 2, and 0.916 for Part 3.

#### Test–retest reliability

Patients in the main analysis repeated the questionnaire twice, 2 weeks apart, and test–retest reliability was assessed by calculating Cohen’s kappa. In the overall study population, the highest kappa coefficients were for P3-4 (0.988), P2-1 (0.971), and P3-5 (0.961), and the lowest were for P1-10 (0.097), P3-9 (0.266), and P1-7 (0.286) (Table 3). Overall, most items in Part 1 had low reliability (except P1-3) and most items in Part 3 had high reliability (except P3-9). Patterns of test–retest reliability differed between the subtypes (Table 3). In patients with GD1, most items in Parts 1 and 2 had generally good reliability, whereas items in Part 3 tended to have lower reliability. In patients with GD2, items in Part 1 had low reliability, items in Part 2 had intermediate reliability, and items in Part 3 had high reliability. In patients with GD3, most items in all three parts had high reliability.

**Table 3:** Test–retest reliability (kappa coefficient) for main analysis

Item	Overall		GD1		GD2		GD3	
	n	Estimate (95% CI)	n	Estimate (95% CI)	n	Estimate (95% CI)	n	Estimate (95% CI)
Part 1								
P1-1	22	0.446 (−0.112, 1)	7	**0.774 (0.774, 0.774)**	9	−0.438 (−1, 1)	6	**0.889 (0.714, 1)**
P1-2	26	0.349 (−1, 1)	8	NA	10	*−0.067 (−1, 1)*	8	**0.842 (0.842, 0.842)**
P1-3	10	**0.828 (0.828, 0.828)**	4	NA	2	NA	4	**0.750 (0.577, 0.923)**
P1-4	27	0.468 (−0.158, 1)	8	NA	10	*−0.067 (−1, 1)*	9	**0.787 (0.561, 1)**
P1-5	25	0.556 (0.269, 0.844)	8	*− 0.111 (−1, 1)*	8	0.481 (−0.355, 1)	9	0.471 (−0.199, 1)
P1-6	28	**0.612 (0.486, 0.738)**	8	**0.800 (0.800, 0.800)**	11	*0.193 (−0.775, 1)*	9	**0.769 (0.769, 0.769)**
P1-7	24	0.286 (−1, 1)	8	**0.778 (0.778, 0.778)**	7	−0.235 (−1, 1)	9	0.542 (0.233, 0.852)
P1-8	26	0.438 (−0.788, 1)	8	0.373 (−1, 1)	9	*0.091 (−0.847, 1)*	9	**0.890 (0.890, 0.890)**
P1-9	23	0.390 (− 0.931, 1)	8	**0.833 (0.833, 0.833)**	7	0.222 (−1, 1)	8	0.517 (−0.179, 1)
P1-10	26	*0.097 (−1, 1)*	8	**0.625 (0.625, 0.625)**	10	−0.231 (−1, 1)	8	0.333 (−0.848, 1)
P1-11	28	0.487 (−1, 1)	8	**0.867 (0.867, 0.867)**	11	*−0.039 (−1, 1)*	9	**0.679 (0.679, 0.679)**
P1-12	24	0.538 (0.155, 0.922)	7	**0.887 (0.705, 1)**	8	*0.132 (−1, 1)*	9	0.518 (−0.365, 1)
P1-13	30	0.444 (−0.388, 1)	8	*0.094 (−0.941, 1)*	13	0.221 (−0.656, 1)	9	**0.780 (0.780, 0.780)**
P1-14	30	0.496 (−0.605, 1)	8	0.576 (−1, 1)	13	0.300 (−0.565, 1)	9	*0.046 (−1, 1)*
P1-15	30	**0.608 (0.331, 0.884)**	8	0.467 (−0.166, 1)	13	0.273 (−1, 1)	9	**0.798 (0.798, 0.798)**
Part 2								
P2-1	29	**0.971 (0.971, 0.971)**	8	**0.857 (0.857, 0.857)**	12	*0.175 (−1, 1)*	9	**0.976 (0.976, 0.976)**
P2-2	29	0.562 (−1, 1)	7	*0 (0, 0)*	13	**0.649 (0.649, 0.649)**	9	0.434 (−1, 1)
P2-3	30	**0.781 (0.781, 0.781)**	8	0.364 (−0.215, 0.943)	13	**0.787 (0.787, 0.787)**	9	**0.799 (0.799, 0.799)**
P2-4	30	**0.612 (0.612, 0.612)**	8	0.298 (−0.500, 1)	13	0.363 (−0.397, 1)	9	**0.917 (0.917, 0.917)**
P2-5	30	**0.746 (0.746, 0.746)**	8	**0.926 (0.926, 0.926)**	13	0.483 (−1, 1)	9	**0.900 (0.900, 0.900)**
P2-6	30	**0.630 (0.630, 0.630)**	8	**0.737 (0.737, 0.737)**	13	0.458 (−0.287, 1)	9	**0.739 (0.204, 1)**
P2-7	30	**0.658 (0.174, 1)**	8	**0.846 (0.846, 0.846)**	13	**0.703 (0.703, 0.703)**	9	0.361 (−1, 1)
P2-8	30	0.582 (−0.003, 1)	8	**0.793 (0.793, 0.793)**	13	**0.680 (0.172, 1)**	9	0.247 (−1, 1)
P2-9	30	0.462 (−1, 1)	8	*0.154 (−1, 1)*	13	0.259 (−1, 1)	9	0.428 (−1, 1)
Part 3								
P3-1	30	**0.842 (0.842, 0.842)**	8	**0.769 (0.769, 0.769)**	13	**0.768 (0.768, 0.768)**	9	**0.967 (0.967, 0.967)**
P3-2	30	**0.683 (0.683, 0.683)**	8	0.580 (−0.107, 1)	13	**0.731 (0.731, 0.731)**	9	**0.749 (0.749, 0.749)**
P3-3	30	**0.868 (0.868, 0.868)**	8	NA	13	**0.786 (0.786, 0.786)**	9	**0.856 (0.856, 0.856)**
P3-4	30	**0.988 (0.988, 0.988)**	8	*0 (−1, 1)*	13	**0.988 (0.988, 0.988)**	9	**0.957 (0.957, 0.957)**
P3-5	30	**0.961 (0.961, 0.961)**	8	**0.760 (0.760, 0.760)**	13	**0.937 (0.937, 0.937)**	9	**0.942 (0.942, 0.942)**
P3-6	29	**0.855 (0.855, 0.855)**	7	NA	13	**0.869 (0.869, 0.869)**	9	0.518 (−1, 1)
P3-7	30	**0.803 (0.803, 0.803)**	8	**0.917 (0.917, 0.917)**	13	**0.692 (0.058, 1)**	9	**0.862 (0.862, 0.862)**
P3-8	30	**0.811 (0.811, 0.811)**	8	**0.609 (0.609, 0.609)**	13	**0.731 (0.731, 0.731)**	9	**0.947 (0.800, 1)**
P3-9	30	0.266 (−1, 1)	8	*0.191 (−0.206, 0.589)*	13	0.248 (−0.665, 1)	9	0.286 (−0.0775, 1)
P3-10	30	**0.659 (0.482, 0.835)**	8	**0.841 (0.600, 1)**	13	0.511 (−0.486, 1)	9	**0.654 (0.160, 1)**
P3-11	30	0.536 (−1, 1)	8	*0.167 (−1, 1)*	13	0.277 (−1, 1)	9	**0.930 (0.930, 0.930)**
P3-12	29	**0.608 (−0.024, 1)**	8	*0 (0, 0)*	12	0.361 (−0.553, 1)	9	**0.696 (0.048, 1)**
P3-13	30	**0.776 (0.776, 0.776)**	8	**0.632 (− 0.154, 1)**	13	**0.736 (0.359, 1)**	9	**0.860 (0.860, 0.860)**
P3-14	30	**0.782 (0.782, 0.782)**	8	*−0.070 (−1, 0.869)*	13	**0.759 (0.286, 1)**	9	**0.658 (0.658, 0.658)**
P3-15	30	**0.753 (0.753, 0.753)**	8	*−0.172 (−1, 1)*	13	**0.845 (0.845, 0.845)**	9	**0.657 (0.012, 1)**
P3-16	27	0.514 (−0.255, 1)	6	**0.848 (0.848, 0.848)**	12	0.470 (−0.605, 1)	9	*0.112 (−1, 1)*

Kappa coefficients > 0.6 are in bold; coefficients < 0.2 are in italics. In general, a kappa coefficient < 0.2 indicates no agreement, 0.2–0.4 indicates minimal agreement, 0.4–0.6 indicates weak agreement, 0.6–0.8 indicates moderate agreement, 0.8–0.9 indicates strong agreement, and > 0.9 indicates almost perfect agreement [29]

CI: confidence interval; GD1/2/3: type 1/2/3 Gaucher disease; NA: not applicable; P: Part

#### Inter-item correlations

**Fig. 1:**
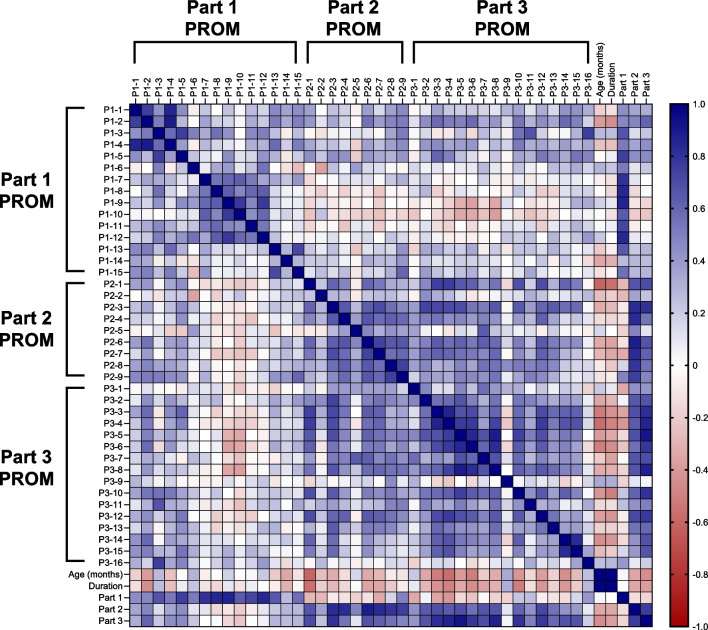
Inter-item correlations in the main analysis of all patients with GD. The magnitude of the correlation coefficients is indicated by the color (as shown on the scale; blue indicates a positive correlation, red indicates a negative correlation). GD: Gaucher disease; P: Part; PROM: patient-reported outcome measure

**Fig. 2:**
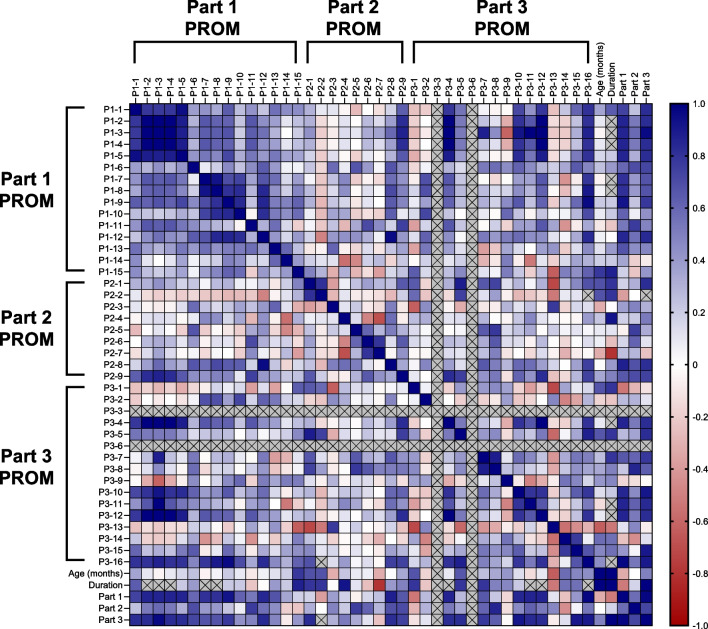
Inter-item correlations in the main analysis of patients with GD1. The magnitude of the correlation coefficients is indicated by the color (as shown on the scale; blue indicates a positive correlation, red indicates a negative correlation). An “X” indicates that there were insufficient data to determine a correlation coefficient. GD1: type 1 Gaucher disease; P: Part; PROM: patient-reported outcome measure

**Fig. 3:**
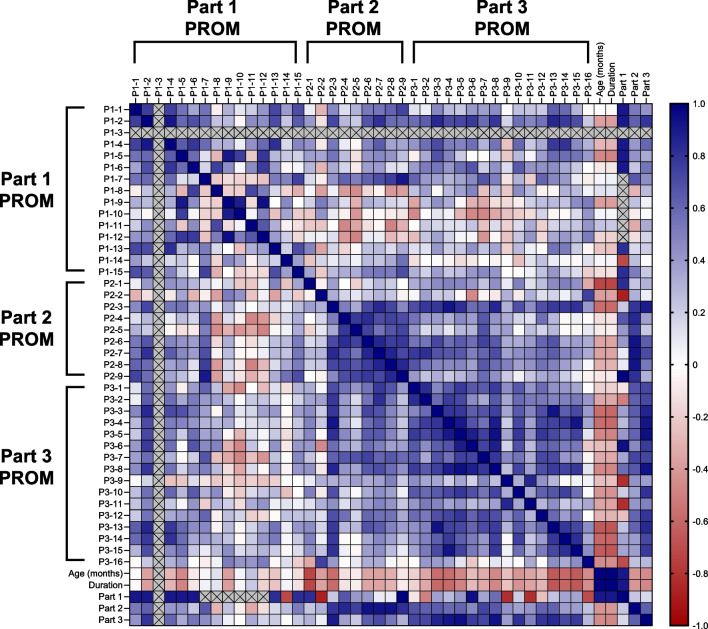
Inter-item correlations in the main analysis of patients with GD2. The magnitude of the correlation coefficients is indicated by the color (as shown on the scale; blue indicates a positive correlation, red indicates a negative correlation). An “X” indicates that there were insufficient data to determine a correlation coefficient. GD2: type 2 Gaucher disease; P: Part; PROM: patient-reported outcome measure

**Fig. 4:**
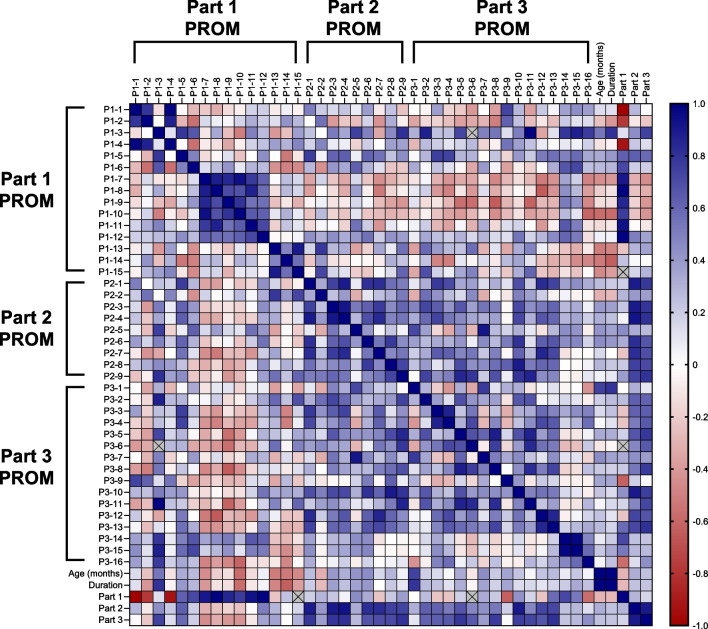
Inter-item correlations in the main analysis of patients with GD3. The magnitude of the correlation coefficients is indicated by the color (as shown on the scale; blue indicates a positive correlation, red indicates a negative correlation). An “X” indicates that there were insufficient data to determine a correlation coefficient. GD3: type 3 Gaucher disease; P: Part; PROM: patient-reported outcome measure

In the overall main survey population, Part 1 Items 8–12 (which cover concerns about risk of Parkinson’s disease, financial burden, effect of budget on therapy, access to expert physicians, and GD problems compared with non-GD health problems) tended to be negatively correlated with items in Parts 2 and 3 regarding specific symptoms (Fig. 1). Most items in Part 2 were positively correlated with items in Part 3. Most items in Parts 2 and 3 were negatively correlated with patient age and duration of disease, primarily in patients with GD2 (Fig. 3); the strongest negative correlation with age and disease duration was for P2-1, which asks about dependence on others. Specific correlation patterns were seen for the different GD types. In GD1, most Part 1 items were negatively correlated with P2-2 (abdomen swelling) and positively correlated with P3-16 (participation in GD patient support group) (Fig. 2). P2-4 (physical weakness) was strongly negatively correlated with P1-14 (all medical concerns were GD related) and P2-7 (GD-related worries), and P3-1 (difficulty hearing) was negatively correlated with P2-3 (fatigue). In GD2, Items P1-8 to P1-12 were negatively correlated with most Part 2 and 3 items, as seen for the overall survey population (Fig. 3). In GD3, Items P1-7 to P1-10 (concerns about risk of cancer, risk of Parkinson’s disease, financial burden, and how budget may affect therapy) were negatively correlated with most Part 3 items (except P3-14 [lack of understanding of GD by public service office] and P3-15 [lack of social support]) (Fig. 4). P1-3 (restricted intimate relationships) was positively correlated with most Part 3 items (except P3-12 [worried or nervous about going out]), and P3-7 (pain in the body) was strongly positively correlated with P2-5 (bone pain).

## Discussion

In this study, we developed and evaluated a GD-specific PROM that can be used for patients with any type of GD as well as for patients from Japan. The PROM consists of Parts 1 and 2 translated from an English questionnaire primarily developed for GD1 [14], plus a newly created Part 3 based on qualitative interviews of Japanese patients with nGD [16]. Part 3 was developed through the collaboration of healthcare professionals who have expertise in GD together with experts in HRQOL questionnaire design. The questions were tailored to Japanese culture and revised as needed in response to patient and caregiver feedback after the pre-test. Analysis of PROM answers from a total of 49 Japanese patients with GD (or their proxies) indicated that the PROM has high internal consistency and test–retest reliability and can be adapted for use in all types of GD. Future use of the PROM is expected to provide insights into the experience of patients with GD, including the extent of burden, factors that contribute to burden, and the effects of medical management and treatment on burden.

Analysis indicated that the PROM has high consistency and test–retest reliability, although there may be some repetition between items. The Cronbach’s alpha was > 0.8 for all three parts, indicating excellent internal consistency. This is supported by the positive inter-item correlations within each part. However, the exceptionally high Cronbach’s alpha for Part 3 (0.916 in the main survey) suggests that there may be some redundancy between items within this part [17]. Additionally, inter-item correlation analysis suggested that most items in Part 2 were positively correlated with items in the newly developed Part 3, which possibly reflects some repetition between these two parts [18]. However, Part 3 had a high level of test–retest reproducibility, indicated by kappa coefficients > 0.60 for 13/16 items. These results suggest that although the current PROM is consistent and reliable, further refinement may be needed for optimization.

In both the pre-test and the main survey, the overall burden was highest in patients with GD2 and lowest in patients with GD1. The higher burden experienced by patients with GD2 may partly relate to their age, as these patients were mainly children whose PROM items were answered by a caregiver as a proxy, whereas all patients with GD1 were adults who completed the PROM themselves. Although obtaining answers directly from patients rather than caregivers would be ideal, this is not always possible in real-life circumstances, such as when patients are very young children or have severe neurological symptoms. Nevertheless, it is not unusual for questions to be answered by proxy for very young patients, such as in a previous study of HRQOL in children with GD1 [13]. In our study, patients with GD3 were intermediate in age, with both children and adults included, and reported an intermediate level of burden. In support of this, we found that older age and longer duration of GD were correlated with a lower level of burden indicated by most PROM items, particularly in Parts 2 and 3, in patients with GD2. These results are consistent with the natural history of neuronopathic GD2, which usually develops in late infancy and progresses rapidly [1, 19]. We speculate that burden may be especially high soon after diagnosis but may decrease with time as treatment stabilizes the patient’s condition and caregivers become more proficient at care.

Chronic fatigue is a well-known symptom of GD and, together with bone pain, contributes to reduced social interaction and quality of life [20]. Despite being a debilitating symptom, fatigue has not been investigated thoroughly in patients with GD, and there is no GD-specific instrument for measuring fatigue [21]. Although case reports suggest that ERT improves chronic fatigue [22–24], exploratory modeling has not supported an association between fatigue and duration of ERT [25]. In the current study, the highest scoring item in Part 3 was P3-13, which refers to feeling tired after hospital visits or treatment. Scores for this item were consistently high across all GD types, indicating that chronic fatigue is an important unmet need for improving HRQOL. Thus, development of a GD-specific assessment scale that can be used to monitor the effect of treatment on fatigue would be highly useful.

**Fig. 5:**
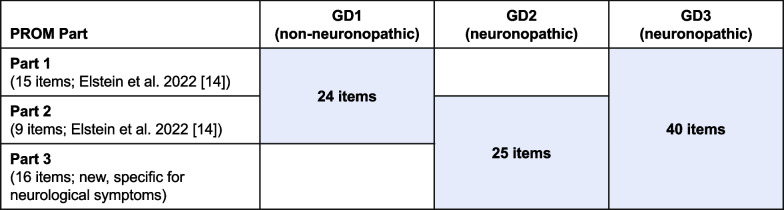
Recommendation for GD type–specific PROM items. GD: Gaucher disease; GD1/2/3: type 1/2/3 Gaucher disease; PROM: patient-reported outcome measure

Regarding the use of this PROM, based on the test–retest reliability and other results, we propose that the PROM can be tailored for use in each type of GD, with Parts 1 and 2 used for GD1 (as initially used by Elstein et al. [14]), Parts 2 and 3 for GD2, and Parts 1, 2, and 3 for GD3 (Fig. 5). The high proportion of incomplete answers to Part 1 for patients with GD2 (all of whom required a proxy) indicates that this section is unlikely to be applicable to these patients. Conversely, as the new Part 3 focuses on neurological symptoms, it is not applicable to patients with GD1. Additionally, before the PROM can be used in practice, a minimal important change in score (or subscores for each part) should be established in the future, which will allow the effects of therapy, as well as changes arising from the natural course of GD, on HRQOL to be evaluated [26, 27].

The limitations of this study include sample sizes that were smaller than planned, which is often a challenge when recruiting patients with a rare disease. Moreover, recruitment of ~ 50 patients represents a relatively large proportion of the estimated ~ 200 patients with GD in Japan [3]. For some items, a limited amount of data could be collected, which may reflect the difficulties some patients experienced in answering these items. However, because of their young age and/or neurological symptoms, all patients with GD2 and one-third of patients with GD3 required a caregiver to complete the PROM on their behalf, which may not accurately reflect the patients’ perception of burden. Despite this, proxy reports are recognized as useful and necessary in cases where the patient is too young or too cognitively impaired to complete a PROM themselves [28]. Furthermore, it is important to compare the disease burden of GD with the burden of other lysosomal diseases that present with similar neurological symptoms, such as Niemann-Pick disease type C and mucopolysaccharidosis. Therefore, research is needed to examine whether this scale, developed as a GD-specific PROM, is comparable to scales developed for other diseases or if it could be modified for use in other diseases. Finally, additional refinement of the PROM may be needed to minimize repetition, and translated versions of Part 3 need to be validated in populations outside Japan. Although this study demonstrated that Part 3 of the Japanese PROM has good reliability, future studies are needed, including construct validation based on hypothesis verification methods, determination of minimal important changes in scores, and cognitive debriefing for the English-language version. Our study has demonstrated the linguistic validity of the Japanese translations of Parts 1 and 2, as well as validated the newly created Japanese Part 3. Once Part 3 has been linguistically and cross-culturally validated in English (or other languages), the PROM can be used for multinational comparison of HRQOL and burden in patients with GD.

## Conclusion

By combining an existing GD-specific PROM with a new set of items aimed at nGD, we have developed a flexible and reliable PROM that can be used to assess patient burden in all types of GD.

## Methods

### Study design

This was a cross-sectional observational study conducted in Japan that comprised three stages. Stage 1 consisted of qualitative interviews with patients with nGD to determine which domains in available GD PROMs were relevant to the development of a Japan-specific PROM; Stage 1 results have been published [16]. Stage 2, conducted May 10–20, 2021, consisted of pre-testing of the PROM (created based on Stage 1) in patients with GD (or their caregivers), followed by 1:1 interviews to collect feedback on the applicability and difficulties in completing the PROM. Stage 3, conducted from October 17 to December 31, 2021, consisted of the main PROM survey of patients (or their caregivers). In Stage 3, participants completed the same survey twice, 2 weeks apart. The study was conducted in accordance with the ethical principles that have their origin in the Declaration of Helsinki, the Guidelines for Good Pharmacoepidemiology Practices, and all applicable laws and regulations. The informed consent form and study protocol were approved by Osaka University Clinical Research Review Committee (No. 20342). Written informed consent was required from all participants or their legal representative before starting the study. Data collected during the study were de-identified and anonymized.

### Study population

Recruitment was conducted in partnership with a patient association group in Japan (Association of Gaucher Disease Patients in Japan) and by referral from leading doctors (main survey only). Eligible patients had a confirmed diagnosis of GD (types 1, 2, or 3) by a physician and had been treated for GD; undiagnosed patients were not included. For patients < 16 years old, a caregiver (≥ 20 years old) participated on behalf of the patient and answered items if patients had difficulty answering themselves, such as for patients who had severe neurological symptoms. Participants were excluded if they were not fluent in Japanese. Participants were included in the study only once (i.e. in the pre-test or in the main test, not both). Each participant who completed the questionnaire received a gift card for 2000 Japanese yen.

### Questionnaires

The questionnaire consisted of three parts (Additional file 1: Table S1; Additional file 2: Table S2). Parts 1 and 2 were translated into Japanese from the previously published PROM [14], with linguistic validation involving two specialist GD physicians. Part 1 consisted of 15 items, each answered on a 1–5-point scale; the score was decreased by 1 to create a 0–4-point scale and then multiplied by 2.5, resulting in a 0–10-point scale with a maximum (highest burden) total of 150 points. Part 2 consisted of nine items, each on a scale of 0–10 points, with a maximum total of 90 points. Part 3 initially consisted of 15 newly developed items, each on a 0–10-point scale, for a maximum total score of 150 points. However, in response to feedback from patients in the pre-test stage, one item (“Over the past week, have you had any difficulty swallowing food or speaking?”) was divided into two items for the main survey (“Over the past week, have you had any difficulty swallowing food?” and “Over the past week, have you had any difficulty speaking?”). Therefore, there were 16 items in the main survey questionnaire, with a maximum total score of 160 points. All items in this report refer to the final PROM numbering.

### Data collection

Paper questionnaires (pre-test and main survey) were mailed from Osaka University to participants through the patient association group. In the pre-test survey (Stage 2), participants completed the questionnaire once prior to an online interview. In the main survey (Stage 3), participants completed each round of the questionnaire and returned the completed questionnaires and consent form to Osaka University. When the paper survey was returned, study personnel checked that the answers were valid before manually entering the data into a preconfigured electronic spreadsheet. Demographic and clinical characteristic questions were included as screening questions in the survey.

### Interviews

In the pre-testing, patients and their caregivers participated in a 1:1 interview to provide feedback on the applicability and difficulties in completing the pre-test questionnaire.

Interviews were conducted in Japanese by independent, qualified interviewers (IQVIA Solutions Japan K.K.); Y. Koto was also present for some interviews.

### Statistical analysis

Consistent with the study objectives, sample sizes were based primarily on the expected feasibility of recruiting patients with a rare disease. The planned sample size for pre-testing was 20 patients. Based on a feasibility assessment and the unbalanced distribution of GD types in Japan, a sample of 50 patients was planned for the main survey. A two-sided 95% confidence interval (CI), assuming 60% of patients with GD (all types) in Japan have had at least one symptom in the past 6 months, would equate to 13.86% precision, and a sample size of 40–50 patients with GD was calculated as sufficient.

Descriptive analysis was performed on all collected data for the overall study population and for GD type subgroups. Total scores for each part (Parts 1, 2, and 3) and scores for individual items were analyzed for the overall study population and for GD type subgroups. Inter-item correlation coefficients were calculated for the pre-test and the main survey. Content consistency was evaluated by Cronbach's alpha, calculated using all cases with complete answers, for both the pre-test and the main survey. Test–retest reliability was evaluated by Cohen's kappa (with CI) using responses from the two rounds of questionnaires in the main survey.

Data that were not collected were not imputed. No sensitivity analysis was conducted. Multiplicity was not applicable for this study, and no adjustments for confounding variables or bias were performed. The R, Version 4.0.2 was used for analysis of the pre-test and main survey questionnaires (R Foundation for Statistical Computing, Vienna, Austria).

### Supplementary Information

**Additional file 1**. **Table S1** Topics covered by PROM items in English. Parts 1 and 2 were from the previously published PROM for GD1 (Elstein D, et al. Orphanet J Rare Dis. 2022;17:9), whereas Part 3 was newly developed.

**Additional file 2**. **Table S2** Topics covered by PROM items in Japanese. Parts 1 and 2 were translated into Japanese from the previously published PROM for GD1 (Elstein D, et al. Orphanet J Rare Dis. 2022;17:9), whereas Part 3 was newly developed.

**Additional file 3**. **Table S3** PROM scores for individual items.

**Additional file 4**. **Fig. S1** Correlations between PROM items in the overall pre-test analysis population. The magnitude of the correlation coefficients is indicated by the color (as shown on the scale; blue indicates a positive correlation, red indicates a negative correlation). An “X” indicates that there were insufficient data to determine a correlation coefficient. Item numbers refer to the final questionnaire used in the main survey. In Part 3, Item 3 (“Over the past week, have you had any difficulty swallowing food or speaking?”) in the pre-test was split into two items in the main survey (P3-3: “Over the past week, have you had any difficulty swallowing food?”; P3-4: “Over the past week, have you had any difficulty speaking?”). P: Part; PROM: patient-reported outcome measure.

## Data Availability

The data generated and/or analyzed during the current study are not openly available due to reasons of sensitivity and are available from the first and corresponding authors with permission from Takeda on reasonable request.

## Abbreviations

CI: Confidence interval
ERT: Enzyme replacement therapy
GD: Gaucher disease
GD1/2/3: Type 1/2/3 GD
HRQOL: Health-related quality of life
MIM: Mendelian Inheritance in Man
nGD: Neuronopathic GD
PROM: Patient-reported outcome measure
SD: Standard deviation

## References

[1] Nalysnyk L, Rotella P, Simeone JC, Hamed A, Weinreb N (2017). Gaucher disease epidemiology and natural history: a comprehensive review of the literature. Hematology.

[2] Japan Society of Congenital Metabolic Disorders. Gaucher's disease clinical practice guidelines. Tokyo, Japan: Shindan To Chiryo Sha; 2021.

[3] Koto Y, Sakai N, Lee Y, Kakee N, Matsuda J, Tsuboi K (2021). Prevalence of patients with lysosomal storage disorders and peroxisomal disorders: a nationwide survey in Japan. Mol Genet Metab.

[4] Stirnemann J, Belmatoug N, Camou F, Serratrice C, Froissart R, Caillaud C (2017). A review of Gaucher disease pathophysiology, clinical presentation and treatments. Int J Mol Sci.

[5] Charrow J, Andersson HC, Kaplan P, Kolodny EH, Mistry P, Pastores G (2000). The Gaucher registry: demographics and disease characteristics of 1698 patients with Gaucher disease. Arch Intern Med.

[6] Ida H (2012). Gaucher disease/inherited metabolic disease syndrome part 2 (2nd edition). Nippon Rinsho (Supplement).

[7] Zimran A, Belmatoug N, Bembi B, Deegan P, Elstein D, Fernandez-Sasso D (2018). Demographics and patient characteristics of 1209 patients with Gaucher disease: descriptive analysis from the Gaucher Outcome Survey (GOS). Am J Hematol.

[8] Gupta N, Oppenheim IM, Kauvar EF, Tayebi N, Sidransky E (2011). Type 2 Gaucher disease: phenotypic variation and genotypic heterogeneity. Blood Cells Mol Dis.

[9] Roshan Lal T, Seehra GK, Steward AM, Poffenberger CN, Ryan E, Tayebi N (2020). The natural history of type 2 Gaucher disease in the 21st century: a retrospective study. Neurology.

[10] Slade A, Isa F, Kyte D, Pankhurst T, Kerecuk L, Ferguson J (2018). Patient reported outcome measures in rare diseases: a narrative review. Orphanet J Rare Dis.

[11] Snyder CF, Aaronson NK (2009). Use of patient-reported outcomes in clinical practice. Lancet.

[12] Alioto AG, Gomez R, Moses J, Paternostro J, Packman S, Packman W (2020). Quality of life and psychological functioning of pediatric and young adult patients with Gaucher disease, type 1. Am J Med Genet A.

[13] Remor E, Baldellou A (2018). Health-related quality of life in children and adolescents living with Gaucher disease and their parents. Health Psychol Behav Med.

[14] Elstein D, Belmatoug N, Deegan P, Göker-Alpan Ö, Hughes DA, Schwartz IVD (2022). Development and validation of Gaucher disease type 1 (GD1)-specific patient-reported outcome measures (PROMs) for clinical monitoring and for clinical trials. Orphanet J Rare Dis.

[15] Elstein D, Klemen M, Panter C, Bonner N, Johnson C, Zimran A (2019). Gaucher disease (GD)-specific patient-reported outcome (PRO) measures for clinical monitoring and for clinical trials. Mol Genet Metab.

[16] Koto Y, Narita A, Noto S, Ono M, Hamada AL, Sakai N (2022). Qualitative analysis of patient interviews on the burden of neuronopathic Gaucher disease in Japan. Orphanet J Rare Dis.

[17] Streiner DL (2003). Starting at the beginning: an introduction to coefficient alpha and internal consistency. J Pers Assess.

[18] Piedmont RL, Michalos AC (2014). Inter-item correlations. Encyclopedia of quality of life and well-being research.

[19] Sidransky E (2012). Gaucher disease: insights from a rare Mendelian disorder. Discov Med.

[20] Hayes RP, Grinzaid KA, Duffey EB, Elsas LJ (1998). The impact of Gaucher disease and its treatment on quality of life. Qual Life Res.

[21] Zion YC, Pappadopulos E, Wajnrajch M, Rosenbaum H (2016). Rethinking fatigue in Gaucher disease. Orphanet J Rare Dis.

[22] Erdal İ, Yıldız Y, Önal G, Aktepe OH, Düzgün SA, Sağlam A (2023). Splenic gaucheroma leading to incidental diagnosis of Gaucher disease in a 46-year-old man with a rare GBA mutation: a case report. Endocr Metab Immune Disord Drug Targets.

[23] Niederau C, vom Dahl S, Häussinger D (1998). First long-term results of imiglucerase therapy of type 1 Gaucher disease. Eur J Med Res.

[24] Vujosevic S, Medenica S, Vujicic V, Dapcevic M, Bakic N, Yang R (2019). Gaucher disease in Montenegro—genotype/phenotype correlations: five cases report. World J Clin Cases.

[25] Wyatt K, Henley W, Anderson L, Anderson R, Nikolaou V, Stein K (2012). The effectiveness and cost-effectiveness of enzyme and substrate replacement therapies: a longitudinal cohort study of people with lysosomal storage disorders. Health Technol Assess.

[26] Devji T, Carrasco-Labra A, Guyatt G (2021). Mind the methods of determining minimal important differences: three critical issues to consider. Evid Based Ment Health.

[27] Terwee CB, Peipert JD, Chapman R, Lai J-S, Terluin B, Cella D (2021). Minimal important change (MIC): a conceptual clarification and systematic review of MIC estimates of PROMIS measures. Qual Life Res.

[28] Varni JW, Limbers CA, Burwinkle TM (2007). Parent proxy-report of their children’s health-related quality of life: an analysis of 13,878 parents’ reliability and validity across age subgroups using the PedsQL 4.0 Generic Core Scales. Health Qual Life Outcomes.

[29] McHugh ML (2012). Interrater reliability: the kappa statistic. Biochem Med.

