# Supplementary material for: Development and evaluation of a patient-reported outcome measure specific for Gaucher disease with or without neurological symptoms in Japan
Source: Orphanet J Rare Dis. 2024 Jan 5;19:11. doi: 10.1186/s13023-023-02996-9 (PMC10770997; doi:10.1186/s13023-023-02996-9)
Supplement: Supplementary file 1 — Additional file 1. Table S1 Topics covered by PROM items in English. Parts 1 and 2 were from the previously published PROM for GD1 (Elstein D, et al. Orphanet J Rare Dis. 2022;17:9), whereas Part 3 was newly developed. [file 13023_2023_2996_MOESM1_ESM.pdf]

Additional file 1: Table S1 Topics covered by PROM items in English. Parts 1 and 2 were from the previously published PROM for GD1 (Elstein D, et al. Orphanet J Rare Dis. 2022;17:9), whereas Part 3 was newly developed

Item	Topic
Part 1	Over the past month:
P1-1	restricted education/job
P1-2	restricted activities with friends
P1-3	restricted intimate relationships
P1-4	restricted hobbies/leisure activities
P1-5	concerned about emotional burden to others
P1-6	concerned about risk of bone disease
P1-7	concerned about risk of cancers
P1-8	concerned about risk of Parkinson's disease
P1-9	concerned about financial burden
P1-10	concerned that budget may affect therapy
P1-11	concerned about access to expert physician
P1-12	non-GD problems compared with GD problems
P1-13	general health improvement because of GD medication
P1-14	all medical concerns were GD related
P1-15	current medication treated GD-related concerns
Part 2	Over the past week:
P2-1	GD-related dependence on others
P2-2	GD-related abdomen swelling
P2-3	GD-related fatigue
P2-4	GD-related physical weakness
P2-5	GD-related bone pain
P2-6	GD-related depression

P2-7	GD-related worries
P2-8	future with GD
P2-9	satisfaction with GD medication
Part 3	Over the past week:
P3-1	difficulty hearing
P3-2	difficulty seeing
P3-3	difficulty swallowing food
P3-4	difficulty speaking
P3-5	involuntary movement or difficulty moving your arms or legs
P3-6	epileptic seizures interfering with daily life
P3-7	pain in the body
P3-8	feel anxious about above symptoms (No. 1-7)
P3-9	memory loss
P3-10	difficulty exercising, studying, or working compared with other persons
P3-11	feel anxious about continuing GD treatment
P3-12	feel worried or nervous about going out
	Up to this point:
P3-13	felt physically tired after visiting the hospital or treatment
P3-14	felt lack of understanding of GD by public service office
P3-15	lack of social support
P3-16	participation in GD patient support group

GD: Gaucher disease; GD1: type 1 GD; PROM: patient-reported outcome measure
