# Supplementary material for: Development and evaluation of a patient-reported outcome measure specific for Gaucher disease with or without neurological symptoms in Japan
Source: Orphanet J Rare Dis. 2024 Jan 5;19:11. doi: 10.1186/s13023-023-02996-9 (PMC10770997; doi:10.1186/s13023-023-02996-9)
Supplement: Supplementary file 2 — Additional file 2. Table S2 Topics covered by PROM items in Japanese. Parts 1 and 2 were translated into Japanese from the previously published PROM for GD1 (Elstein D, et al. Orphanet J Rare Dis. 2022;17:9), whereas Part 3 was newly developed. [file 13023_2023_2996_MOESM2_ESM.pdf]

Additional file 2: Table S2 Topics covered by PROM items in Japanese. Parts 1 and 2 were translated into Japanese from the previously published PROM for GD1 (Elstein D, et al. Orphanet J Rare Dis. 2022;17:9), whereas Part 3 was newly developed

**Item**	質問項目
Part 1	過去 1 ヵ月の下記の程度を問う質問：
P1-1	学業・仕事の制限
P1-2	友人付き合いの制限
P1-3	配偶者・パートナーとの関係の制限
P1-4	趣味や余暇活動の制限
P1-5	他者の精神的な負担になる心配
P1-6	骨症状の心配
P1-7	癌のリスクの心配
P1-8	パーキンソン病のリスクの心配
P1-9	経済的負担の心配
P1-10	最良の治療が受けられない心配
P1-11	専門医への相談についての心配
P1-12	GD 以外の心配
P1-13	治療薬による健康状態の改善
P1-14	健康上の懸念と GD
P1-15	治療薬と症状の管理
Part 2	過去 1 週間の下記の程度を問う質問：
P2-1	他者からの支援
P2-2	腹部症状
P2-3	疲労感

P2-4	体力の衰え
P2-5	骨の痛み
P2-6	気分の落ち込み
P2-7	疾患についての悩み
P2-8	将来について
P2-9	薬物療法への満足
Part 3	過去 1 週間の下記の程度を問う質問：
P3-1	耳の聞こえにくさ
P3-2	目の見えにくさ
P3-3	食事の飲み込みにくさ
P3-4	しゃべりにくさ
P3-5	手足の動かしにくさ
P3-6	てんかんの発作
P3-7	体の痛み
P3-8	1～7 に挙げたような症状に対する不安
P3-9	物忘れ
P3-10	運動や学習・仕事での難しさ
P3-11	治療の継続についての不安
P3-12	外出への不安
	これまでの下記の程度を問う質問：
P3-13	通院による体の疲れ
P3-14	行政サービスの窓口の担当者の理解不足
P3-15	社会支援の不足
P3-16	患者会での情報交換

GD: Gaucher disease; GD1: type 1 GD; PROM: patient-reported outcome measure
