# Supplementary material for: Development and evaluation of a patient-reported outcome measure specific for Gaucher disease with or without neurological symptoms in Japan
Source: Orphanet J Rare Dis. 2024 Jan 5;19:11. doi: 10.1186/s13023-023-02996-9 (PMC10770997; doi:10.1186/s13023-023-02996-9)
Supplement: Supplementary file 3 — Additional file 3. Table S3 PROM scores for individual items. [file 13023_2023_2996_MOESM3_ESM.pdf]

Additional file 3: Table S3 PROM scores for individual items

Item[a]	Pre-test				Main analysis			
	**GD1 (N=3)**	**GD2 (N=6)**	**GD3 (N=7)**	**Overall (N=16)**	**GD1 (N=9)**	**GD2 (N=13)**	**GD3 (N=11)**	**Overall (N=33)**
Part 1								
P1-1								
Mean (SD)	0.833 (1.44)	5.63 (5.15)	4.58 (4.31)	4.04 (4.27)	1.67 (2.80)	4.50 (3.07)	4.38 (4.38)	3.52 (3.55)
Median	0	6.25	2.50	2.50	0	5.00	3.75	2.50
(min, max)	(0, 2.50)	(0, 10.0)	(0, 10.0)	(0, 10.0)	(0, 7.50)	(0, 10.0)	(0, 10.0)	(0, 10.0)
Not collected, n (%)	0 (0)	2 (33.3)	1 (14.3)	3 (18.8)	0 (0)	3 (23.1)	3 (27.3)	6 (18.2)
P1-2								
Mean (SD)	0 (0)	3.13 (3.75)	4.29 (5.35)	3.04 (4.40)	0.556 (1.67)	3.64 (4.52)	2.50 (3.12)	2.33 (3.53)
Median	0	2.50	0	0	0	0	2.50	0
(min, max)	(0, 0)	(0, 7.50)	(0, 10.0)	(0, 10.0)	(0, 5.00)	(0, 10.0)	(0, 10.0)	(0, 10.0)

Item[a]	Pre-test				Main analysis			
	GD1 (N=3)	GD2 (N=6)	GD3 (N=7)	Overall (N=16)	GD1 (N=9)	GD2 (N=13)	GD3 (N=11)	Overall (N=33)
Not collected, n (%)	0 (0)	2 (33.3)	0 (0)	2 (12.5)	0 (0)	2 (15.4)	1 (9.1)	3 (9.1)
P1-3								
Mean (SD)	0 (NA)	0 (0)	1.25 (2.50)	0.625 (1.77)	1.67 (4.08)	0 (0)	1.50 (2.24)	1.17 (2.81)
Median	0	0	0	0	0	0	0	0
(min, max)	(0, 0)	(0, 0)	(0, 5.00)	(0, 5.00)	(0, 10.0)	(0, 0)	(0, 5.00)	(0, 10.0)
Not collected, n (%)	2 (66.7)	3 (50.0)	3 (42.9)	8 (50.0)	3 (33.3)	9 (69.2)	6 (54.5)	18 (54.5)
P1-4								
Mean (SD)	0.833 (1.44)	5.63 (5.15)	3.93 (4.53)	3.75 (4.36)	0.833 (2.50)	3.25 (4.09)	3.86 (4.38)	2.75 (3.90)
Median	0	6.25	2.50	2.50	0	1.25	2.50	0
(min, max)	(0, 2.50)	(0, 10.0)	(0, 10.0)	(0, 10.0)	(0, 7.50)	(0, 10.0)	(0, 10.0)	(0, 10.0)

Item[a]	Pre-test				Main analysis			
	GD1 (N=3)	GD2 (N=6)	GD3 (N=7)	Overall (N=16)	GD1 (N=9)	GD2 (N=13)	GD3 (N=11)	Overall (N=33)
Not collected, n (%)	0 (0)	2 (33.3)	0 (0)	2 (12.5)	0 (0)	3 (23.1)	0 (0)	3 (9.1)
P1-5								
Mean (SD)	0 (0)	1.25 (1.77)	2.92 (4.01)	1.82 (3.18)	0.833 (1.77)	1.67 (2.50)	3.41 (3.22)	2.07 (2.76)
Median	0	1.25	1.25	0	0	0	2.50	0
(min, max)	(0, 0)	(0, 2.50)	(0, 10.0)	(0, 10.0)	(0, 5.00)	(0, 7.50)	(0, 10.0)	(0, 10.0)
Not collected, n (%)	0 (0)	4 (66.7)	1 (14.3)	5 (31.3)	0 (0)	4 (30.8)	0 (0)	4 (12.1)
P1-6								
Mean (SD)	6.67 (2.89)	6.25 (3.23)	4.29 (3.13)	5.36 (3.08)	6.94 (3.91)	4.32 (3.37)	3.64 (3.23)	4.84 (3.65)
Median	5.00	6.25	5.00	5.00	10.0	5.00	2.50	5.00
(min, max)	(5.00, 10.0)	(2.50, 10.0)	(0, 10.0)	(0, 10.0)	(0, 10.0)	(0, 10.0)	(0, 10.0)	(0, 10.0)

Item[a]	Pre-test				Main analysis			
	GD1 (N=3)	GD2 (N=6)	GD3 (N=7)	Overall (N=16)	GD1 (N=9)	GD2 (N=13)	GD3 (N=11)	Overall (N=33)
Not collected, n (%)	0 (0)	2 (33.3)	0 (0)	2 (12.5)	0 (0)	2 (15.4)	0 (0)	2 (6.1)
P1-7								
Mean (SD)	5.00 (NA)	0.833 (1.44)	0.714 (1.89)	1.14 (2.05)	1.11 (2.20)	0.833 (2.50)	2.00 (3.07)	1.34 (2.59)
Median	5.00	0	0	0	0	0	1.25	0
(min, max)	(5.00, 5.00)	(0, 2.50)	(0, 5.00)	(0, 5.00)	(0, 5.00)	(0, 7.50)	(0, 10.0)	(0, 10.0)
Not collected, n (%)	2 (66.7)	3 (50.0)	0 (0)	5 (31.3)	0 (0)	4 (30.8)	1 (9.1)	5 (15.2)
P1-8								
Mean (SD)	5.00 (7.07)	1.25 (1.44)	2.14 (2.25)	2.31 (2.97)	2.78 (4.41)	2.50 (3.75)	2.75 (3.62)	2.68 (3.78)
Median	5.00	1.25	2.50	2.50	0	0	1.25	0
(min, max)	(0, 10.0)	(0, 2.50)	(0, 5.00)	(0, 10.0)	(0, 10.0)	(0, 10.0)	(0, 10.0)	(0, 10.0)

Item[a]	Pre-test				Main analysis			
	GD1 (N=3)	GD2 (N=6)	GD3 (N=7)	Overall (N=16)	GD1 (N=9)	GD2 (N=13)	GD3 (N=11)	Overall (N=33)
Not collected, n (%)	1 (33.3)	2 (33.3)	0 (0)	3 (18.8)	0 (0)	4 (30.8)	1 (9.1)	5 (15.2)
P1-9								
Mean (SD)	5.00 (0)	3.33 (5.77)	2.86 (2.25)	3.33 (3.08)	2.50 (3.06)	0.714 (1.89)	3.61 (3.33)	2.40 (3.02)
Median	5.00	0	2.50	3.75	0	0	5.00	0
(min, max)	(5.00, 5.00)	(0, 10.0)	(0, 5.00)	(0, 10.0)	(0, 7.50)	(0, 5.00)	(0, 10.0)	(0, 10.0)
Not collected, n (%)	1 (33.3)	3 (50.0)	0 (0)	4 (25.0)	0 (0)	6 (46.2)	2 (18.2)	8 (24.2)
P1-10								
Mean (SD)	0 (NA)	0 (0)	1.79 (1.89)	1.04 (1.67)	1.39 (1.82)	0.682 (1.62)	1.67 (3.31)	1.21 (2.28)
Median	0	0	2.50	0	0	0	0	0
(min, max)	(0, 0)	(0, 0)	(0, 5.00)	(0, 5.00)	(0, 5.00)	(0, 5.00)	(0, 10.0)	(0, 10.0)

Item[a]	Pre-test				Main analysis			
	GD1 (N=3)	GD2 (N=6)	GD3 (N=7)	Overall (N=16)	GD1 (N=9)	GD2 (N=13)	GD3 (N=11)	Overall (N=33)
Not collected, n (%)	2 (66.7)	2 (33.3)	0 (0)	4 (25.0)	0 (0)	2 (15.4)	2 (18.2)	4 (12.1)
P1-11								
Mean (SD)	2.50 (3.54)	2.50 (2.89)	1.79 (2.38)	2.12 (2.47)	1.11 (2.20)	0.682 (1.62)	2.50 (4.03)	1.45 (2.87)
Median	2.50	2.50	0	0	0	0	0	0
(min, max)	(0, 5.00)	(0, 5.00)	(0, 5.00)	(0, 5.00)	(0, 5.00)	(0, 5.00)	(0, 10.0)	(0, 10.0)
Not collected, n (%)	1 (33.3)	2 (33.3)	0 (0)	3 (18.8)	0 (0)	2 (15.4)	0 (0)	2 (6.1)
P1-12								
Mean (SD)	10.0 (0)	1.88 (3.75)	2.14 (2.67)	3.27 (4.00)	5.31 (4.32)	2.78 (3.84)	4.77 (3.25)	4.29 (3.78)
Median	10.0	0	0	0	5.00	0	5.00	5.00
(min, max)	(10.0, 10.0)	(0, 7.50)	(0, 5.00)	(0, 10.0)	(0, 10.0)	(0, 10.0)	(0, 10.0)	(0, 10.0)

Item[a]	Pre-test				Main analysis			
	GD1 (N=3)	GD2 (N=6)	GD3 (N=7)	Overall (N=16)	GD1 (N=9)	GD2 (N=13)	GD3 (N=11)	Overall (N=33)
Not collected, n (%)	1 (33.3)	2 (33.3)	0 (0)	3 (18.8)	1 (11.1)	4 (30.8)	0 (0)	5 (15.2)
P1-13								
Mean (SD)	4.17 (1.44)	4.00 (2.85)	3.21 (2.38)	3.67 (2.29)	2.50 (2.50)	3.46 (2.61)	4.32 (2.52)	3.48 (2.57)
Median	5.00	5.00	2.50	2.50	2.50	2.50	2.50	2.50
(min, max)	(2.50, 5.00)	(0, 7.50)	(0, 7.50)	(0, 7.50)	(0, 5.00)	(0, 7.50)	(2.50, 10.0)	(0, 10.0)
Not collected, n (%)	0 (0)	1 (16.7)	0 (0)	1 (6.3)	0 (0)	0 (0)	0 (0)	0 (0)
P1-14								
Mean (SD)	4.17 (1.44)	9.00 (2.24)	4.64 (3.36)	6.00 (3.38)	4.44 (2.08)	6.54 (2.98)	4.09 (2.02)	5.15 (2.65)
Median	5.00	10.0	5.00	5.00	5.00	5.00	5.00	5.00
(min, max)	(2.50, 5.00)	(5.00, 10.0)	(0, 10.0)	(0, 10.0)	(0, 7.50)	(0, 10.0)	(0, 7.50)	(0, 10.0)

Item[a]	Pre-test				Main analysis			
	**GD1 (N=3)**	**GD2 (N=6)**	**GD3 (N=7)**	**Overall (N=16)**	**GD1 (N=9)**	**GD2 (N=13)**	**GD3 (N=11)**	**Overall (N=33)**
Not collected, n (%)	0 (0)	1 (16.7)	0 (0)	1 (6.3)	0 (0)	0 (0)	0 (0)	0 (0)
P1-15								
Mean (SD)	3.33 (1.44)	4.50 (3.26)	2.86 (2.25)	3.50 (2.46)	1.67 (1.25)	2.50 (2.28)	4.09 (3.02)	2.80 (2.48)
Median	2.50	5.00	2.50	2.50	2.50	2.50	5.00	2.50
(min, max)	(2.50, 5.00)	(0, 7.50)	(0, 7.50)	(0, 7.50)	(0, 2.50)	(0, 7.50)	(0, 10.0)	(0, 10.0)
Not collected, n (%)	0 (0)	1 (16.7)	0 (0)	1 (6.3)	0 (0)	0 (0)	0 (0)	0 (0)
Part 2								
P2-1								
Mean (SD)	0 (0)	10.0 (0)	4.43 (4.24)	5.69 (4.67)	0.889 (1.36)	8.92 (2.29)	4.09 (3.73)	5.12 (4.25)
Median	0	10.0	2.00	8.00	0	10.0	4.00	4.00
(min, max)	(0, 0)	(10.0, 10.0)	(0, 10.0)	(0, 10.0)	(0, 3.00)	(3.00, 10.0)	(0, 10.0)	(0, 10.0)

Item[a]	Pre-test				Main analysis			
	GD1 (N=3)	GD2 (N=6)	GD3 (N=7)	Overall (N=16)	GD1 (N=9)	GD2 (N=13)	GD3 (N=11)	Overall (N=33)
Not collected, n (%)	0 (0)	0 (0)	0 (0)	0 (0)	0 (0)	0 (0)	0 (0)	0 (0)
P2-2								
Mean (SD)	0 (0)	1.20 (2.68)	2.43 (4.16)	1.53 (3.23)	0.125 (0.354)	1.23 (2.62)	1.45 (1.81)	1.03 (2.01)
Median	0	0	0	0	0	0	0	0
(min, max)	(0, 0)	(0, 6.00)	(0, 9.00)	(0, 9.00)	(0, 1.00)	(0, 8.00)	(0, 5.00)	(0, 8.00)
Not collected, n (%)	0 (0)	1 (16.7)	0 (0)	1 (6.3)	1 (11.1)	0 (0)	0 (0)	1 (3.0)
P2-3								
Mean (SD)	4.33 (1.15)	7.50 (3.79)	2.86 (2.19)	4.50 (3.16)	3.22 (1.86)	6.08 (3.25)	3.82 (3.79)	4.55 (3.31)
Median	5.00	9.00	2.00	4.50	3.00	7.00	2.00	5.00
(min, max)	(3.00, 5.00)	(2.00, 10.0)	(0, 6.00)	(0, 10.0)	(0, 5.00)	(0, 10.0)	(0, 10.0)	(0, 10.0)

Item[a]	Pre-test				Main analysis			
	**GD1 (N=3)**	**GD2 (N=6)**	**GD3 (N=7)**	**Overall (N=16)**	**GD1 (N=9)**	**GD2 (N=13)**	**GD3 (N=11)**	**Overall (N=33)**
Not collected, n (%)	0 (0)	2 (33.3)	0 (0)	2 (12.5)	0 (0)	0 (0)	0 (0)	0 (0)
P2-4								
Mean (SD)	4.00 (1.73)	4.25 (2.99)	3.00 (3.79)	3.57 (3.08)	2.44 (1.88)	4.69 (3.66)	4.27 (3.58)	3.94 (3.29)
Median	5.00	5.00	2.00	4.00	3.00	5.00	4.00	3.00
(min, max)	(2.00, 5.00)	(0, 7.00)	(0, 10.0)	(0, 10.0)	(0, 5.00)	(0, 10.0)	(0, 10.0)	(0, 10.0)
Not collected, n (%)	0 (0)	2 (33.3)	0 (0)	2 (12.5)	0 (0)	0 (0)	0 (0)	0 (0)
P2-5								
Mean (SD)	2.67 (3.06)	3.25 (4.72)	0.571 (1.13)	1.79 (2.97)	4.11 (3.14)	2.85 (3.36)	1.55 (2.02)	2.76 (3.00)
Median	2.00	1.50	0	0	3.00	0	1.00	2.00
(min, max)	(0, 6.00)	(0, 10.0)	(0, 3.00)	(0, 10.0)	(0, 8.00)	(0, 9.00)	(0, 6.00)	(0, 9.00)

Item[a]	Pre-test				Main analysis			
	GD1 (N=3)	GD2 (N=6)	GD3 (N=7)	Overall (N=16)	GD1 (N=9)	GD2 (N=13)	GD3 (N=11)	Overall (N=33)
Not collected, n (%)	0 (0)	2 (33.3)	0 (0)	2 (12.5)	0 (0)	0 (0)	0 (0)	0 (0)
P2-6								
Mean (SD)	0 (0)	5.00 (3.56)	1.00 (1.26)	2.00 (2.89)	1.67 (1.87)	4.85 (3.41)	3.09 (2.51)	3.39 (2.99)
Median	0	6.00	0.500	0	1.00	5.00	2.00	3.00
(min, max)	(0, 0)	(0, 8.00)	(0, 3.00)	(0, 8.00)	(0, 5.00)	(0, 10.0)	(0, 6.00)	(0, 10.0)
Not collected, n (%)	0 (0)	2 (33.3)	1 (14.3)	3 (18.8)	0 (0)	0 (0)	0 (0)	0 (0)
P2-7								
Mean (SD)	2.00 (2.65)	5.75 (4.35)	2.43 (3.64)	3.29 (3.77)	2.11 (2.37)	3.77 (3.14)	2.00 (3.10)	2.73 (2.97)
Median	1.00	6.50	1.00	1.50	1.00	5.00	0	1.00
(min, max)	(0, 5.00)	(0, 10.0)	(0, 10.0)	(0, 10.0)	(0, 5.00)	(0, 10.0)	(0, 8.00)	(0, 10.0)

Item[a]	Pre-test				Main analysis			
	GD1 (N=3)	GD2 (N=6)	GD3 (N=7)	Overall (N=16)	GD1 (N=9)	GD2 (N=13)	GD3 (N=11)	Overall (N=33)
Not collected, n (%)	0 (0)	2 (33.3)	0 (0)	2 (12.5)	0 (0)	0 (0)	0 (0)	0 (0)
P2-8								
Mean (SD)	3.67 (2.31)	5.00 (4.08)	2.86 (3.67)	3.64 (3.43)	2.78 (2.22)	3.77 (3.47)	3.64 (3.41)	3.45 (3.09)
Median	5.00	5.00	2.00	4.00	2.00	4.00	2.00	3.00
(min, max)	(1.00, 5.00)	(0, 10.0)	(0, 10.0)	(0, 10.0)	(0, 5.00)	(0, 10.0)	(0, 9.00)	(0, 10.0)
Not collected, n (%)	0 (0)	2 (33.3)	0 (0)	2 (12.5)	0 (0)	0 (0)	0 (0)	0 (0)
P2-9								
Mean (SD)	0.333 (0.577)	3.80 (3.42)	3.57 (3.31)	3.00 (3.16)	1.22 (1.72)	2.54 (3.38)	2.55 (2.77)	2.18 (2.79)
Median	0	3.00	3.00	2.00	0	0	2.00	2.00
(min, max)	(0, 1.00)	(0, 9.00)	(0, 10.0)	(0, 10.0)	(0, 5.00)	(0, 9.00)	(0, 10.0)	(0, 10.0)

Item[a]	Pre-test				Main analysis			
	**GD1 (N=3)**	**GD2 (N=6)**	**GD3 (N=7)**	**Overall (N=16)**	**GD1 (N=9)**	**GD2 (N=13)**	**GD3 (N=11)**	**Overall (N=33)**
Not collected, n (%)	0 (0)	1 (16.7)	0 (0)	1 (6.3)	0 (0)	0 (0)	0 (0)	0 (0)
Part 3								
P3-1								
Mean (SD)	NA	5.00 (4.08)	1.57 (3.74)	2.82 (4.05)	2.33 (2.74)	2.85 (2.91)	2.45 (3.62)	2.58 (3.03)
Median (min, max)	NA	5.00 (0, 10.0)	0 (0, 10.0)	0 (0, 10.0)	1.00 (0, 7.00)	4.00 (0, 8.00)	0 (0, 10.0)	0 (0, 10.0)
Not collected, n (%)	3 (100)	2 (33.3)	0 (0)	5 (31.3)	0 (0)	0 (0)	0 (0)	0 (0)
P3-2								
Mean (SD)	NA	6.75 (4.72)	0.667 (1.21)	3.10 (4.25)	1.00 (1.66)	3.77 (3.37)	2.64 (2.84)	2.64 (2.96)
Median (min, max)	NA	8.50 (0, 10.0)	0 (0, 3.00)	0.500 (0, 10.0)	0 (0, 5.00)	5.00 (0, 8.00)	2.00 (0, 8.00)	2.00 (0, 8.00)

Item[a]	Pre-test				Main analysis			
	GD1 (N=3)	GD2 (N=6)	GD3 (N=7)	Overall (N=16)	GD1 (N=9)	GD2 (N=13)	GD3 (N=11)	Overall (N=33)
Not collected, n (%)	3 (100)	2 (33.3)	1 (14.3)	6 (37.5)	0 (0)	0 (0)	0 (0)	0 (0)
P3-3[b]								
Mean (SD)	NA	7.50 (5.00)	3.29 (4.64)	4.82 (5.00)	0 (0)	5.92 (4.79)	2.45 (4.13)	3.15 (4.47)
Median (min, max)	NA	10.0 (0, 10.0)	1.00 (0, 10.0)	2.00 (0, 10.0)	0 (0, 0)	9.00 (0, 10.0)	0 (0, 10.0)	0 (0, 10.0)
Not collected, n (%)	3 (100)	2 (33.3)	0 (0)	5 (31.3)	0 (0)	0 (0)	0 (0)	0 (0)
P3-4[b]								
Mean (SD)	–	–	–	–	0.111 (0.333)	6.38 (4.65)	2.55 (3.88)	3.39 (4.44)
Median (min, max)	–	–	–	–	0 (0, 1.00)	10.0 (0, 10.0)	1.00 (0, 10.0)	0 (0, 10.0)

Item[a]	Pre-test				Main analysis			
	**GD1 (N=3)**	**GD2 (N=6)**	**GD3 (N=7)**	**Overall (N=16)**	**GD1 (N=9)**	**GD2 (N=13)**	**GD3 (N=11)**	**Overall (N=33)**
Not collected, n (%)	–	–	–	–	0 (0)	0 (0)	0 (0)	0 (0)
P3-5								
Mean (SD)	NA	8.20 (3.49)	3.57 (4.54)	5.50 (4.62)	1.22 (1.99)	6.46 (4.43)	2.91 (3.59)	3.85 (4.17)
Median (min, max)	NA	10.0 (2.00, 10.0)	2.00 (0, 10.0)	6.00 (0, 10.0)	0 (0, 5.00)	10.0 (0, 10.0)	2.00 (0, 10.0)	2.00 (0, 10.0)
Not collected, n (%)	3 (100)	1 (16.7)	0 (0)	4 (25.0)	0 (0)	0 (0)	0 (0)	0 (0)
P3-6								
Mean (SD)	NA	7.50 (3.08)	2.14 (3.93)	4.62 (4.41)	0 (0)	5.38 (4.81)	1.45 (3.21)	2.69 (4.21)
Median (min, max)	NA	8.50 (2.00, 10.0)	0 (0, 10.0)	5.00 (0, 10.0)	0 (0, 0)	8.00 (0, 10.0)	0 (0, 10.0)	0 (0, 10.0)

Item[a]	Pre-test				Main analysis			
	GD1 (N=3)	GD2 (N=6)	GD3 (N=7)	Overall (N=16)	GD1 (N=9)	GD2 (N=13)	GD3 (N=11)	Overall (N=33)
Not collected, n (%)	3 (100)	0 (0)	0 (0)	3 (18.8)	1 (11.1)	0 (0)	0 (0)	1 (3.0)
P3-7								
Mean (SD)	NA	4.33 (4.04)	0.857 (1.07)	1.90 (2.69)	2.11 (2.93)	4.31 (3.82)	1.64 (2.29)	2.82 (3.28)
Median (min, max)	NA	5.00 (0, 8.00)	0 (0, 2.00)	1.00 (0, 8.00)	0 (0, 8.00)	4.00 (0, 10.0)	1.00 (0, 6.00)	2.00 (0, 10.0)
Not collected, n (%)	3 (100)	3 (50.0)	0 (0)	6 (37.5)	0 (0)	0 (0)	0 (0)	0 (0)
P3-8								
Mean (SD)	NA	7.00 (2.45)	3.00 (3.51)	4.45 (3.64)	1.44 (1.67)	5.15 (3.83)	3.55 (3.98)	3.61 (3.67)
Median (min, max)	NA	6.50 (5.00, 10.0)	1.00 (0, 10.0)	5.00 (0, 10.0)	2.00 (0, 5.00)	5.00 (0, 10.0)	1.00 (0, 10.0)	2.00 (0, 10.0)

Item[a]	Pre-test				Main analysis			
	GD1 (N=3)	GD2 (N=6)	GD3 (N=7)	Overall (N=16)	GD1 (N=9)	GD2 (N=13)	GD3 (N=11)	Overall (N=33)
Not collected, n (%)	3 (100)	2 (33.3)	0 (0)	5 (31.3)	0 (0)	0 (0)	0 (0)	0 (0)
P3-9								
Mean (SD)	NA	3.33 (2.89)	1.86 (2.85)	2.30 (2.79)	2.56 (1.81)	1.15 (2.19)	3.55 (3.30)	2.33 (2.67)
Median (min, max)	NA	5.00 (0, 5.00)	1.00 (0, 8.00)	1.50 (0, 8.00)	2.00 (0, 5.00)	0 (0, 5.00)	3.00 (0, 10.0)	2.00 (0, 10.0)
Not collected, n (%)	3 (100)	3 (50.0)	0 (0)	6 (37.5)	0 (0)	0 (0)	0 (0)	0 (0)
P3-10								
Mean (SD)	NA	7.75 (2.63)	4.14 (4.38)	5.45 (4.11)	2.67 (3.43)	7.31 (3.92)	3.82 (3.22)	4.88 (4.01)
Median (min, max)	NA	8.00 (5.00, 10.0)	3.00 (0, 10.0)	5.00 (0, 10.0)	1.00 (0, 10.0)	10.0 (0, 10.0)	3.00 (0, 10.0)	4.00 (0, 10.0)

Item[a]	Pre-test				Main analysis			
	GD1 (N=3)	GD2 (N=6)	GD3 (N=7)	Overall (N=16)	GD1 (N=9)	GD2 (N=13)	GD3 (N=11)	Overall (N=33)
Not collected, n (%)	3 (100)	2 (33.3)	0 (0)	5 (31.3)	0 (0)	0 (0)	0 (0)	0 (0)
P3-11								
Mean (SD)	NA	3.40 (3.13)	2.57 (3.60)	2.92 (3.29)	1.11 (2.20)	2.08 (2.87)	2.45 (3.39)	1.94 (2.86)
Median (min, max)	NA	5.00 (0, 6.00)	2.00 (0, 10.0)	2.00 (0, 10.0)	0 (0, 5.00)	0 (0, 8.00)	1.00 (0, 10.0)	0 (0, 10.0)
Not collected, n (%)	3 (100)	1 (16.7)	0 (0)	4 (25.0)	0 (0)	0 (0)	0 (0)	0 (0)
P3-12								
Mean (SD)	NA	6.80 (4.32)	4.57 (2.76)	5.50 (3.50)	0.556 (1.67)	5.42 (3.90)	3.55 (4.01)	3.41 (3.90)
Median (min, max)	NA	9.00 (0, 10.0)	3.00 (2.00, 9.00)	5.50 (0, 10.0)	0 (0, 5.00)	6.50 (0, 10.0)	1.00 (0, 10.0)	1.00 (0, 10.0)

Item[a]	Pre-test				Main analysis			
	GD1 (N=3)	GD2 (N=6)	GD3 (N=7)	Overall (N=16)	GD1 (N=9)	GD2 (N=13)	GD3 (N=11)	Overall (N=33)
Not collected, n (%)	3 (100)	1 (16.7)	0 (0)	4 (25.0)	0 (0)	1 (7.7)	0 (0)	1 (3.0)
P3-13								
Mean (SD)	NA	6.50 (2.38)	5.50 (3.67)	5.90 (3.11)	5.11 (3.02)	5.54 (3.89)	5.73 (3.50)	5.48 (3.44)
Median (min, max)	NA	7.50 (3.00, 8.00)	4.50 (2.00, 10.0)	6.00 (2.00, 10.0)	5.00 (1.00, 10.0)	7.00 (0, 10.0)	6.00 (1.00, 10.0)	6.00 (0, 10.0)
Not collected, n (%)	3 (100)	2 (33.3)	1 (14.3)	6 (37.5)	0 (0)	0 (0)	0 (0)	0 (0)
P3-14								
Mean (SD)	NA	9.00 (1.41)	1.75 (2.36)	5.78 (4.21)	1.89 (1.83)	5.85 (4.14)	2.36 (2.46)	3.61 (3.54)
Median (min, max)	NA	10.0 (7.00, 10.0)	1.00 (0, 5.00)	7.00 (0, 10.0)	2.00 (0, 5.00)	7.00 (0, 10.0)	1.00 (0, 6.00)	3.00 (0, 10.0)

Item[a]	Pre-test				Main analysis			
	GD1 (N=3)	GD2 (N=6)	GD3 (N=7)	Overall (N=16)	GD1 (N=9)	GD2 (N=13)	GD3 (N=11)	Overall (N=33)
Not collected, n (%)	3 (100)	1 (16.7)	3 (42.9)	7 (43.8)	0 (0)	0 (0)	0 (0)	0 (0)
P3-15								
Mean (SD)	NA	4.60 (4.22)	3.50 (3.11)	4.11 (3.59)	1.78 (1.92)	4.54 (3.93)	3.09 (3.70)	3.30 (3.50)
Median	NA	3.00	3.50	3.00	2.00	6.00	1.00	2.00
(min, max)		(0, 10.0)	(0, 7.00)	(0, 10.0)	(0, 5.00)	(0, 10.0)	(0, 10.0)	(0, 10.0)
Not collected, n (%)	3 (100)	1 (16.7)	3 (42.9)	7 (43.8)	0 (0)	0 (0)	0 (0)	0 (0)
P3-16								
Mean (SD)	NA	1.00 (1.41)	3.00 (2.31)	2.17 (2.17)	3.14 (3.76)	2.25 (2.09)	4.60 (3.31)	3.28 (3.06)
Median	NA	0	3.00	2.50	2.00	2.00	5.00	2.00
(min, max)		(0, 3.00)	(0, 6.00)	(0, 6.00)	(0, 10.0)	(0, 6.00)	(0, 10.0)	(0, 10.0)

Item[a]	Pre-test				Main analysis			
	GD1 (N=3)	GD2 (N=6)	GD3 (N=7)	Overall (N=16)	GD1 (N=9)	GD2 (N=13)	GD3 (N=11)	Overall (N=33)
Not collected, n (%)	3 (100)	1 (16.7)	0 (0)	4 (25.0)	2 (22.2)	1 (7.7)	1 (9.1)	4 (12.1)

[a]Item numbers refer to the final questionnaire used in the main survey

[b]In Part 3, Item 3 (“Over the past week, have you had any difficulty swallowing food or speaking?”) in the pre-test was split into two items in the main survey (P3-3: “Over the past week, have you had any difficulty swallowing food?”; P3-4: “Over the past week, have you had any difficulty speaking?”).

GD1/2/3: type 1/2/3 Gaucher disease; max: maximum; min: minimum; NA: not applicable; P: Part; PROM: patient-reported outcome measure; SD: standard deviation
