# Supplementary material for: Development and evaluation of a patient-reported outcome measure specific for Gaucher disease with or without neurological symptoms in Japan
Source: Orphanet J Rare Dis. 2024 Jan 5;19:11. doi: 10.1186/s13023-023-02996-9 (PMC10770997; doi:10.1186/s13023-023-02996-9)

Additional file 4: Fig. S1 Correlations between PROM items in the overall pre-test analysis population. The magnitude of the correlation coefficients is indicated by the color (as shown on the scale; blue indicates a positive correlation, red indicates a negative correlation). An “X” indicates that there were insufficient data to determine a correlation coefficient. Item numbers refer to the final questionnaire used in the main survey. In Part 3, Item 3 (“Over the past week, have you had any difficulty swallowing food or speaking?”) in the pre-test was split into two items in the main survey (P3-3: “Over the past week, have you had any difficulty swallowing food?”; P3-4: “Over the past week, have you had any difficulty speaking?”).

P: Part; PROM: patient-reported outcome measure

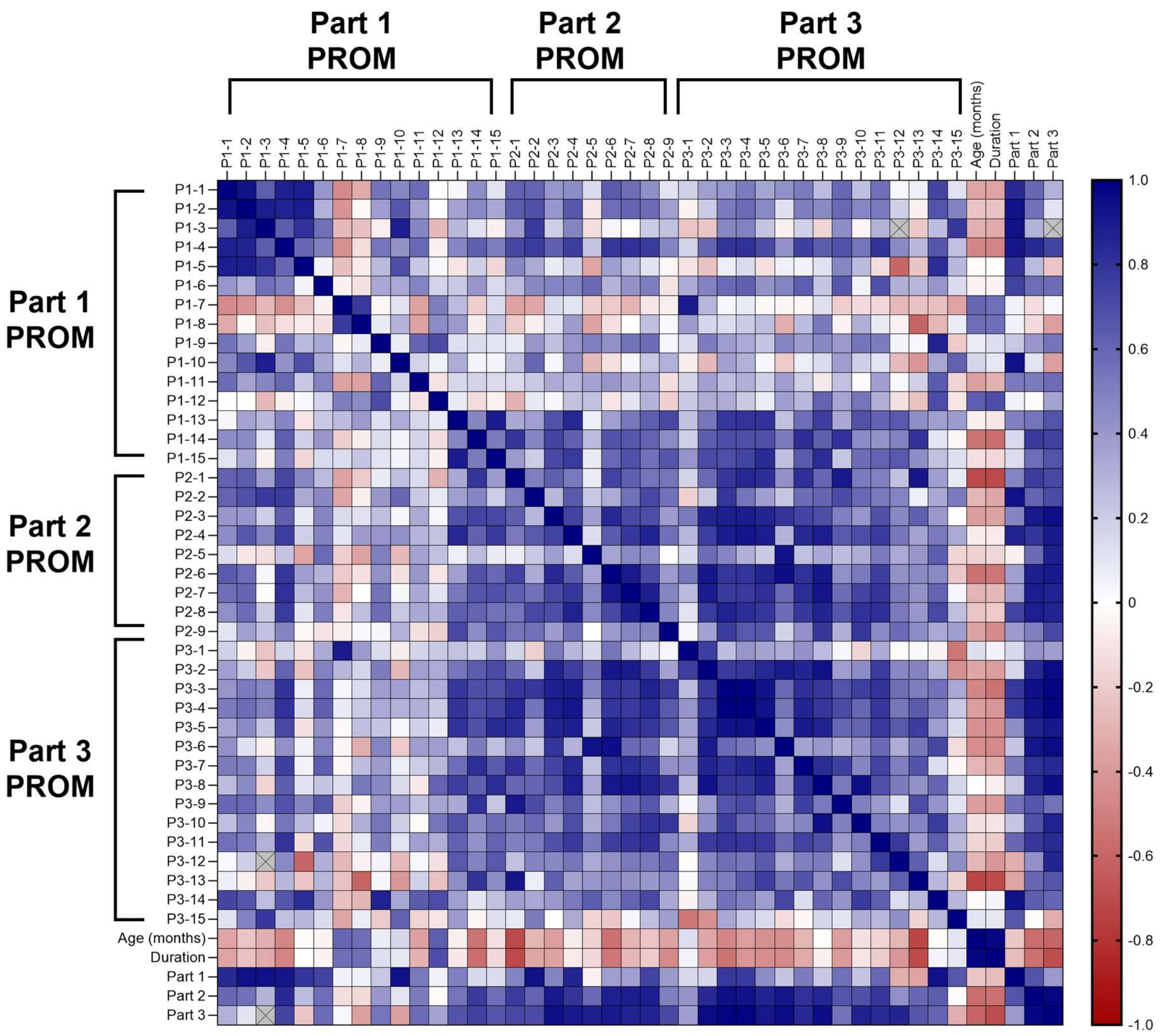

Supplement: Supplementary file 4 — Additional file 4. Fig. S1 Correlations between PROM items in the overall pre-test analysis population. The magnitude of the correlation coefficients is indicated by the color (as shown on the scale; blue indicates a positive correlation, red indicates a negative correlation). An “X” indicates that there were insufficient data to determine a correlation coefficient. Item numbers refer to the final questionnaire used in the main survey. In Part 3, Item 3 (“Over the past week, have you had any difficulty swallowing food or speaking?”) in the pre-test was split into two items in the main survey (P3-3: “Over the past week, have you had any difficulty swallowing food?”; P3-4: “Over the past week, have you had any difficulty speaking?”). P: Part; PROM: patient-reported outcome measure. [file 13023_2023_2996_MOESM4_ESM.pdf]
